# Digital Versus Conventional Splints in Patients With Temporomandibular Disorders: A Systematic Review and Meta-Analysis of Randomized Controlled Trials

**DOI:** 10.7759/cureus.105977

**Published:** 2026-03-27

**Authors:** Fahad W Almansour, Khaled Alshammari, Dhari Mohammed Alhajri, Maryam Bader Alzamanan, Fay Hamed Alduaij, Khaled Walled Abdullah, Mohammad Homoud Alnoumas, Ahmed Abdelaziz

**Affiliations:** 1 Department of Dentistry, Kuwait Ministry of Health, Kuwait City, KWT; 2 Department of Biostatistics, Faculty of Medicine, Al-Azhar University, Cairo, EGY

**Keywords:** cad/cam, digital dentistry, digital occlusal splints, temporomandibular joint (tmj) disorders, tmd

## Abstract

Occlusal splints are widely used as a conservative treatment modality for temporomandibular disorders (TMD). With the increasing integration of digital technologies in dentistry, computer-aided design and manufacturing (CAD/CAM) workflows have emerged. Although digital splints are increasingly adopted in clinical practice, their clinical effectiveness compared with conventionally fabricated splints remains uncertain. Electronic searches were performed in PubMed, Scopus, Web of Science (WOS), and Cochrane Central Register of Controlled Trials (CENTRAL) from database inception to March 2026 to identify randomized controlled trials (RCTs) comparing digital with conventional occlusal splints in patients with TMD. The primary outcome was the mean difference of pain intensity post intervention. Continuous outcomes were analyzed using mean differences (MDs) with 95% confidence intervals (CIs) using a random-effects model. Eight RCTs comprising 390 patients were included. Seven studies reported posttreatment pain scores, showing no significant difference between digital and conventional splints (MD = -0.22; 95% CI = -0.92 to 0.48; p = 0.54; I² = 77.9%). Similarly, no significant differences were observed in limited mouth opening or maximum mouth opening. Patient satisfaction was also comparable between groups. However, digitally fabricated splints were associated with significantly shorter clinical occlusal adjustment time compared with conventional splints (MD = -9.10 minutes; 95% CI = -15.18 to -3.02; p < 0.001). Digital and conventional occlusal splints provide comparable clinical outcomes in patients with TMD with respect to pain relief, mouth opening, and patient satisfaction. However, digitally fabricated splints may offer a practical advantage by reducing clinical adjustment time. Further high-quality trials with standardized outcomes and longer follow-up are warranted.

## Introduction and background

Temporomandibular disorders (TMD) represent a group of musculoskeletal conditions that affect the masticatory muscles, temporomandibular joint, and associated structures [1]. These disorders are a common cause of orofacial pain and functional limitation, with reported prevalence estimates ranging from 5% to 12% in the general population [2,3]. Patients with TMD frequently experience symptoms such as jaw pain, joint sounds, restricted mandibular movement, headaches, and impaired quality of life [4]. Among the various conservative management strategies available, occlusal splints remain one of the most widely used noninvasive treatments [5,6]. Conventional splints are typically fabricated through traditional dental impressions and laboratory processing, and they have been shown to reduce pain, improve jaw function, and decrease parafunctional activity in selected patients [7,8].

Recent advances in digital dentistry have introduced computer-aided design and manufacturing (CAD/CAM) techniques for splint fabrication [9]. Digital splints offer several potential advantages, including improved precision, reproducibility, reduced laboratory variability, and faster manufacturing workflows [10,11]. Early clinical studies have suggested that digitally fabricated splints may provide comparable or potentially improved clinical outcomes compared with conventional splints, particularly with respect to patient comfort, fit, and treatment efficiency [12-14]. However, existing studies have reported heterogeneous findings, and individual trials are often limited by small sample sizes and variations in outcome reporting [12-14].

Despite the growing adoption of digital technologies in dental practice, the comparative effectiveness of digital versus conventional splints in patients with TMD remains uncertain. Therefore, we performed a systematic review and meta-analysis to synthesize the available evidence and evaluate the clinical outcomes associated with digital compared with conventional splint therapy in patients with TMD.

## Review

Materials and methods

This systematic review and meta-analysis was carried out following the guidelines of the Preferred Reporting Items for Systematic Reviews and Meta-Analyses (PRISMA) [15] and the guideline recommendations proposed in the Cochrane Handbook for Systematic Reviews of Interventions [16].

Literature Search and Screening

A comprehensive electronic search was performed in PubMed, Scopus, Web of Science (WOS), and the Cochrane Central Register of Controlled Trials (CENTRAL) from database inception to March 2026 to identify relevant randomized controlled trials (RCTs). The search strategy combined controlled terms related to temporomandibular disorders, occlusal splints, and digital fabrication techniques. The detailed search strategy for each database is provided in Table 1. In addition to the electronic search, the backward and forward citation tracking of the included articles was conducted to identify potentially relevant studies. Titles and abstracts were screened initially, followed by a full-text review of eligible studies to determine their final inclusion. Two reviewers independently performed the screening process, and a third reviewer handled the conflict between the judgements.

**Table 1:** Detailed search strategy according to each database. CENTRAL, Cochrane Central Register of Controlled Trials; TMD, temporomandibular disorder; TMJ, temporomandibular joint; CAD, computer-aided design; CAM, computer-aided manufacturing; SLA, stereolithography

Database	Search term	Field/filters	Date	Result
PubMed	(Temporomandibular disorder OR Temporomandibular disorders OR Temporomandibular joint OR TMD OR TMJ OR Myofascial pain OR Temporomandibular dysfunction OR Craniomandibular disorder) AND (Digital OR CAD/CAM OR CAD-CAM OR Computer aided OR Computer-aided OR 3D printed OR 3D printing OR Additive manufacturing OR Milling OR Milled OR Rapid prototyping OR Stereolithography OR SLA) AND (Splint OR Splints OR Occlusal splint OR Stabilization splint OR Bite guard OR Mouth guard OR Occlusal appliance OR Bite plane)	All fields and English	12th of March 2026	173
Scopus	("Temporomandibular disorder" OR "Temporomandibular disorders" OR "Temporomandibular joint" OR "TMD" OR "TMJ" OR "Myofascial pain" OR "Temporomandibular dysfunction" OR "Craniomandibular disorder") AND ("Digital" OR "CAD/CAM" OR "CAD-CAM" OR "Computer aided" OR "Computer-aided" OR "3D printed" OR "3D printing" OR "Additive manufacturing" OR "Milling" OR "Milled" OR "Rapid prototyping" OR "Stereolithography" OR "SLA") AND ("Splint" OR "Splints" OR "Occlusal splint" OR "Stabilization splint" OR "Bite guard" OR "Mouth guard" OR "Occlusal appliance" OR "Bite plane")	Title, abstract, keyword, and English	12th of March 2026	114
Web of Science	(Temporomandibular disorder OR Temporomandibular disorders OR Temporomandibular joint OR TMD OR TMJ OR Myofascial pain OR Temporomandibular dysfunction OR Craniomandibular disorder) AND (Digital OR CAD/CAM OR CAD-CAM OR Computer aided OR Computer-aided OR 3D printed OR 3D printing OR Additive manufacturing OR Milling OR Milled OR Rapid prototyping OR Stereolithography OR SLA) AND (Splint OR Splints OR Occlusal splint OR Stabilization splint OR Bite guard OR Mouth guard OR Occlusal appliance OR Bite plane)	All fields and English	12th of March 2026	116
CENTRAL	(Temporomandibular disorder OR Temporomandibular disorders OR Temporomandibular joint OR TMD OR TMJ OR Myofascial pain OR Temporomandibular dysfunction OR Craniomandibular disorder) AND (Digital OR CAD/CAM OR CAD-CAM OR Computer aided OR Computer-aided OR 3D printed OR 3D printing OR Additive manufacturing OR Milling OR Milled OR Rapid prototyping OR Stereolithography OR SLA) AND (Splint OR Splints OR Occlusal splint OR Stabilization splint OR Bite guard OR Mouth guard OR Occlusal appliance OR Bite plane)	All fields and English	12th of March 2026	62

Eligibility Criteria

We included all studies with the following criteria: population, patients diagnosed with temporomandibular disorders (TMD); intervention, digitally fabricated occlusal splints; and control, conventionally fabricated occlusal splints; also, the studies had to be randomized controlled trials (RCTs). For randomized controlled trials with a crossover design, only data from the first treatment period prior to crossover were extracted and included in the meta-analysis, in order to avoid potential carryover effects and to maintain consistency with parallel-group trials. When outcomes were reported before the implementation of the crossover phase, these were treated as independent parallel-group comparisons. Data obtained after crossover or from combined treatment periods were not included in the analysis. Studies were excluded if they included patients diagnosed solely with bruxism without a confirmed diagnosis of TMD or if they were review articles, conference abstracts, case reports, or non-randomized studies.

Endpoints

The primary outcome was pain intensity at the last available follow-up, assessed using the Visual Analog Scale (VAS). The VAS is a widely used 10-point scale for pain assessment, where 0 represents no pain, and 10 indicates the worst imaginable pain [17]. Secondary outcomes included maximum and limited mouth opening, patient satisfaction measured using a Likert scale, and clinical occlusal adjustment time, assessed using the T-Scan system [18].

Quality Assessment

We evaluated the quality of the included randomized controlled trials using the Cochrane Risk of Bias tool version 2 (RoB 2) [19]. This tool assesses five domains: randomization process bias, deviations from intended interventions, missing outcome data, outcome measurement, and the selection of the reported results. Each domain was judged as low risk of bias, some concerns, or high risk of bias. Disagreements between reviewers were resolved through discussion with the corresponding author until consensus was reached.

Data Extraction

Data from the included studies were extracted using a standardized Excel sheet (Microsoft Corp., Redmond, WA). The extracted data were as follows: summary and baseline characteristics of the included studies and patients, including study type, country, inclusion and exclusion criteria, sample size, age and gender, assessed outcomes and key findings, risk of bias domains, and the studied outcomes.

Statistical Analysis

Continuous outcomes were extracted as means, standard deviations (SDs), and total sample sizes and pooled using mean differences (MDs) with corresponding 95% confidence intervals (CIs). A DerSimonian-Laird random-effects model was applied to account for potential variability across studies. Statistical heterogeneity was assessed using the I² statistic and Cochran's Q test. Heterogeneity was considered substantial when I² ≥ 50% or when the Q-test p-value was <0.10. When substantial heterogeneity was detected, leave-one-out sensitivity analyses were conducted to evaluate the influence of individual studies on the pooled estimates. All statistical analyses were performed using STATA version 19 MP (StataCorp LLC, College Station, TX).

Results

Literature Search and Study Selection

**Figure 1:**
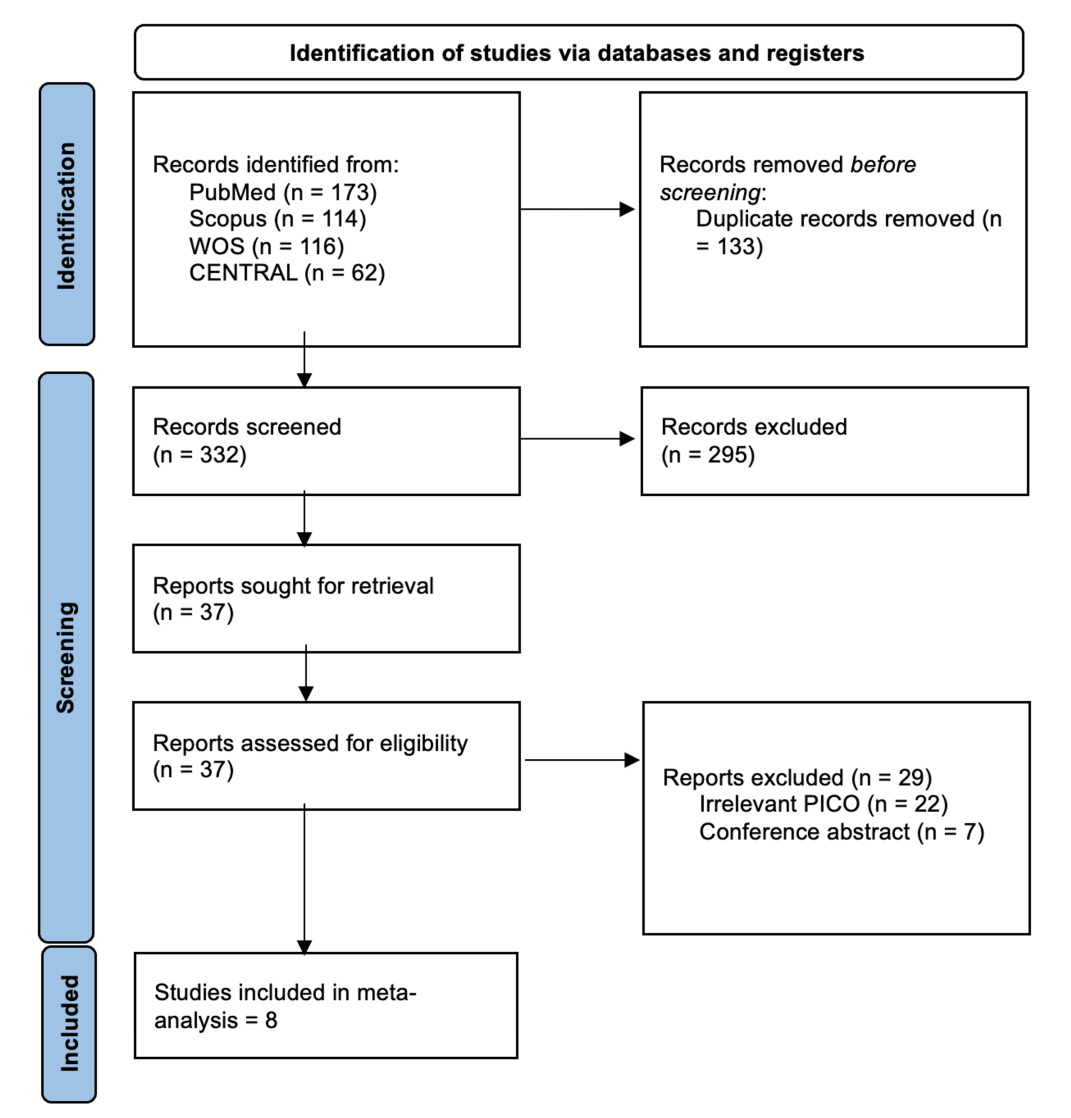
PRISMA flowchart. PRISMA, Preferred Reporting Items for Systematic Reviews and Meta-Analyses; WOS, Web of Science; PICO, population, intervention, control, and outcome; CENTRAL, Cochrane Central Register of Controlled Trials

Our search yielded 465 articles, of which 428 were excluded following title-abstract screening and duplicate removal, leaving 37 for full-text screening. Eight RCTs were finally included in the analysis [11-14,18,20-22]. The selection process is shown in the PRISMA chart (Figure 1).

Study Characteristics and Quality Assessment

The final eight included trials (comprising six parallel-group and wo crossover randomized controlled trials) were conducted between 2016 and 2026 [11-14,18,20-22]. The trials included a total of 390 patients diagnosed with TMD. Patients received either digitally fabricated occlusal splints or conventionally fabricated acrylic occlusal splints. The detailed summary of the included studies and baseline characteristics of the included patients are summarized in Table 2 and the Appendices.

**Table 2:** Summary characteristics of the included studies. CAD, computer-aided design; CAM, computer-aided design manufacturing; SLA, stereolithography; TMD, temporomandibular disorder; DC/TMD, Diagnostic Criteria for Temporomandibular Disorder; TMJ, temporomandibular joint; RCT, randomized controlled trial; PMMA, polymethylmethacrylate; RDC/TMD, Research Diagnostic Criteria for Temporomandibular Disorder; DDWR, disc displacement with reduction; NR, not reported

Study	Country	Time period	Design	Sample size	Intervention criteria	Control criteria	Inclusion criteria	Exclusion criteria
Qin et al., 2025 [11]	China	March 2023 to July 2023	Single-center, parallel RCT	126	Digital 3D-printed stable occlusal splint (intraoral scan, CAD, and SLA 3D-printed with Dental LT Clear resin)	Conventional handmade stable occlusal splint (vacuum-formed shell with self-curing resin)	18-65 years, TMD diagnosis (DC/TMD), sufficient teeth for retention, and no prior TMD treatment	Limited mouth opening preventing impression, obvious periodontal disease, orthodontic treatment, removable dentures, maxillofacial trauma/TMJ surgery history, pregnancy, and severe systemic/mental diseases
Chen et al., 2026 [12]	China	January 2024 to June 2024	RCT	132	Fully digital occlusal splint (intraoral scan, CAD, and CNC milled or 3D-printed)	Conventional occlusal splint	Complete dentition, clinical TMD diagnosis, and adequate oral function	Systemic conditions affecting bone/immune function (osteoporosis and rheumatoid arthritis) and severe parafunctional habits
Abd Elgaleel et al., 2025 [13]	Egypt	November 2025 to December 2025	RCT, crossover	12 (24 splints total)	3D-printed maxillary stabilization splint (DLP, HARZ Labs Dental Clear resin)	Conventional heat-cured acrylic resin (PMMA) maxillary stabilization splint	Female, 20-50 years, physically/psychologically able, good oral hygiene, no prior splint therapy, and Angle Class I	Substantial dental/periodontal disease, prior psychological or occlusal splint therapy, and malocclusion
Hussein et al., 2025 [14]	Egypt	April 2024 to not specified	RCT	20	Digitally fabricated occlusal splint (intraoral scan, CAD, and SLA 3D-printed with Detax Freeprint resin)	Traditional occlusal splint (heat-cured acrylic resin)	Myogenic TMD (RDC/TMD), both genders, 18-45 years, and the same number of original molars	Arthrogenic/disc displacement TMD, acute TMJ injury, dental prostheses/implants, >2 molars missing, acute toothache, cognitive deficits, and medication intake
Pai et al., 2025 [18]	India	Not specified	RCT, crossover	24	Digitally fabricated stabilization splint (intraoral scan, CAD, and 3D-printed with Dental LT Clear resin)	Conventionally fabricated stabilization splint (heat-cured acrylic resin)	20-50 years, pain-related TMD (myalgia or arthralgia) based on DC/TMD, complete permanent dentition, and stable occlusion	TMJ trauma/surgery, systemic arthropathies, ongoing orthodontic/prosthodontic treatment, analgesic use, parafunctional habits, and pregnancy
Algabri et al., 2017 [20]	Egypt	2015-2016	Parallel RCT	30	CAD/CAM stabilization occlusal splint	Conventional stabilization occlusal splint	20-45 years, TMD with painful TMJ click, no functional mouth limitation, TMJ tenderness, and the absence of prior splint/dental disease	Teeth loss affecting splint support, systemic diseases (rheumatoid arthritis and osteoporosis), analgesic/muscle relaxant use, and the absence of DDWR on MRI
Berntsen et al., 2018 [21]	Norway	2016-2017	Parallel RCT	14	CAD-CAM additive-manufactured stabilization splints	Conventional manufactured stabilization splints	TMD diagnosis myalgia	Severe jaw functional limitations, severe somatic symptoms, depression, anxiety, removable dentures, recent trauma, dentoalveolar pathology, and ongoing TMD treatment
Pho Duc et al., 2016 [22]	Germany	NR	Parallel RCT	32	CAD/CAM milled stabilization splint (high-density acrylic block)	Conventional stabilization splint (heat-cured acrylic resin)	20-50 years, self-reported pain in masticatory muscles/TMJ for ≥3 months, and aggravated by function	Physical conditions interfering with pain, recent trauma, orthodontic treatment, splint use, medication use (muscle relaxants), systemic diseases, and pregnancy

**Figure 2:**
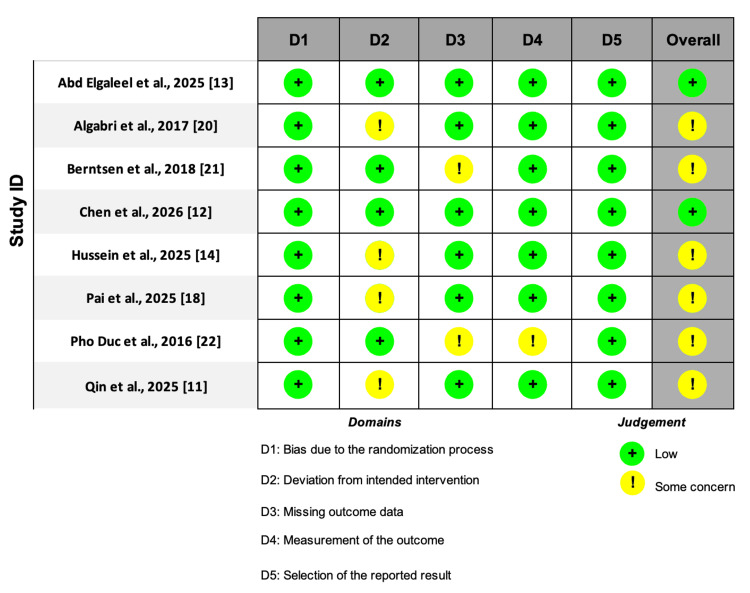
Risk of bias assessment using ROB 2 tool for RCTs. RoB 2, Risk of Bias version 2; RCTs, randomized controlled trials

The quality of the eight RCTs was assessed using the Cochrane Risk of Bias version 2 (RoB 2), which showed that most of the studies were moderate-to-high quality of evidence, as shown in Figure 2.

Primary Outcome

**Figure 3:**
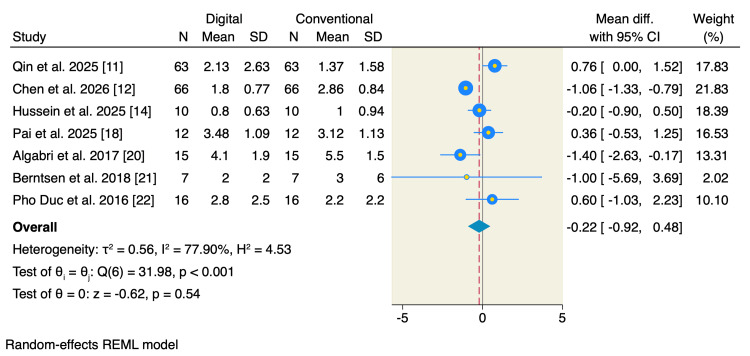
Random-effects model for pain scores at the last follow-up. SD, standard deviation; CI, confidence interval; REML, restricted maximum likelihood

**Figure 4:**
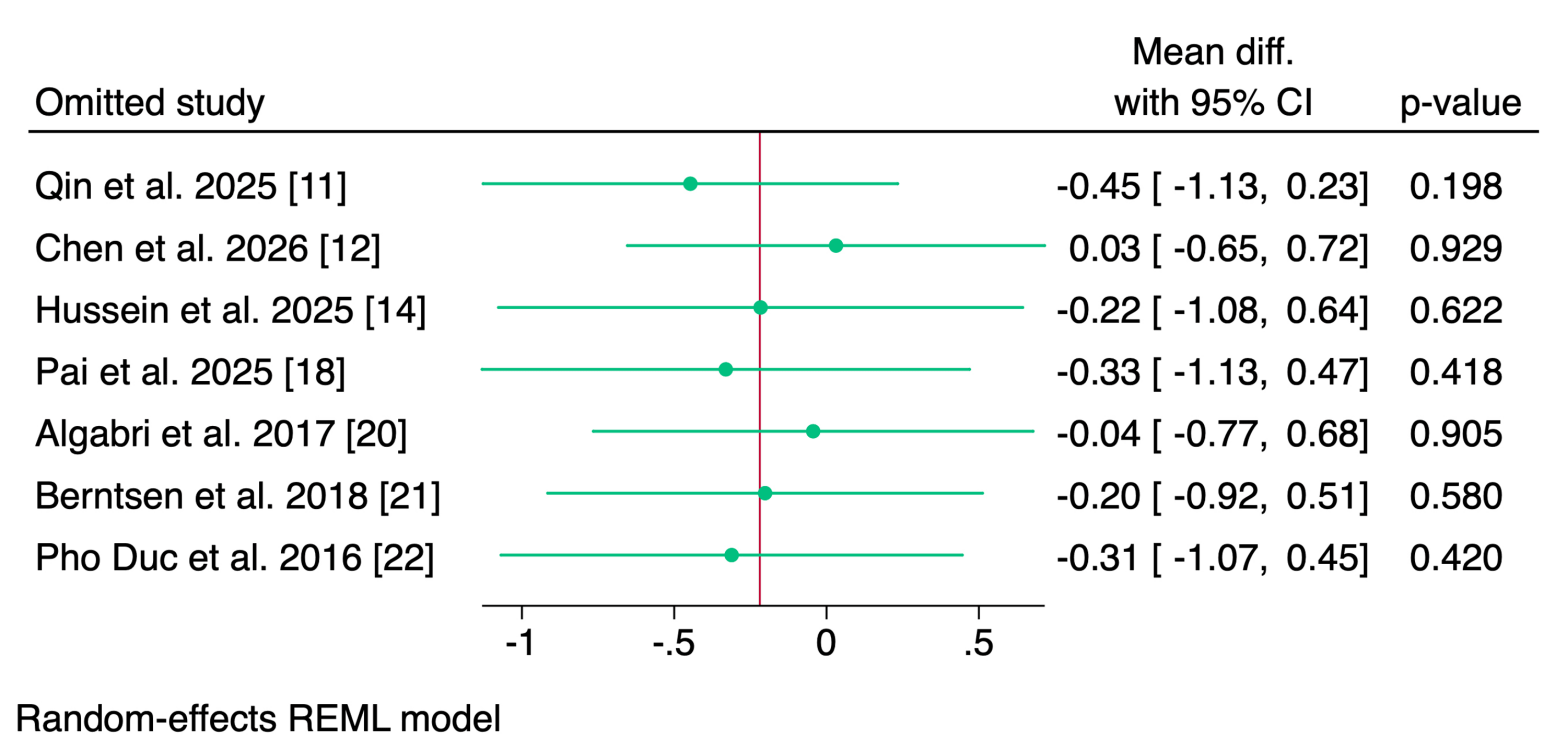
Leave-one-out sensitivity analysis of pain scores. CI, confidence interval; REML, restricted maximum likelihood

The postoperative pain scores at the last follow-up were reported by seven studies, of which the pooled analysis showed no significant difference between digitally and conventionally fabricated splints (MD = -0.22; 95% CI: -0.92 to 0.48; p = 0.54; I² = 77.90%; p < 0.001) (Figure 3). Leave-one-out sensitivity analysis showed that no single study had a disproportional effect on the pooled MD (Figure 4).

Secondary Outcomes

**Figure 5:**
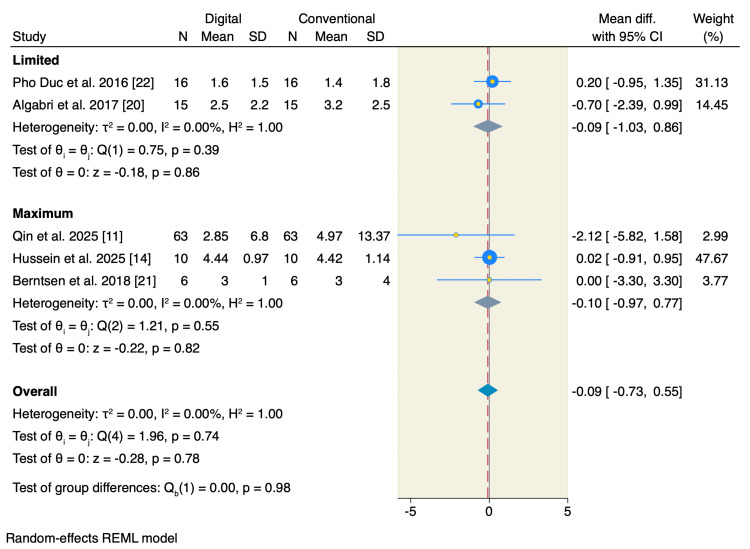
Random-effects model for mouth opening. SD, standard deviation; CI, confidence interval; REML, restricted maximum likelihood

**Figure 6:**
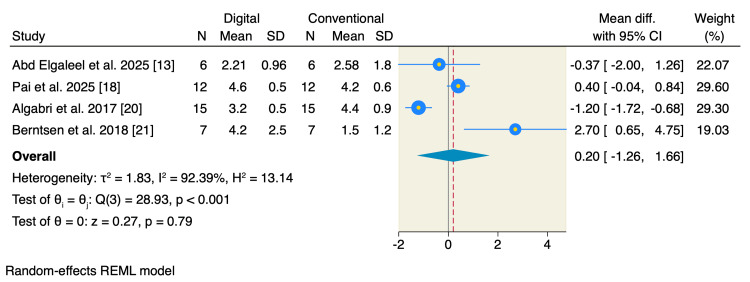
Random-effects model for patients' satisfaction. SD, standard deviation; CI, confidence interval; REML, restricted maximum likelihood

**Figure 7:**
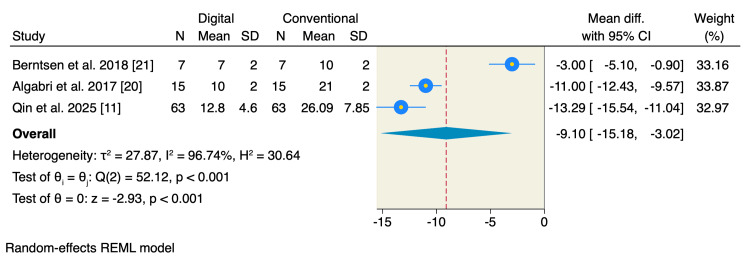
Random-effects model for occlusal adjustment time. SD, standard deviation; CI, confidence interval; REML, restricted maximum likelihood

Five studies reported mouth opening, of which the pooled estimate showed no significant difference between the two studied groups in terms of limited mouth opening (MD = -0.09; 95% CI: -1.03 to 0.86; p = 0.86; I² = 0.00%) or maximum mouth opening (MD = -0.10; 95% CI: -0.97 to 0.77; p = 0.82; I² = 0.00%) (Figure 5). Only four studies reported the scale of the patient's satisfaction, of which the pooled estimate showed no significant difference between the two studied groups (MD = 0.20; 95% CI: -1.26 to 1.66; p = 0.79; I² = 92.39%) (Figure 6). On the other hand, patients allocated to the digitally fabricated occlusal splints had less time needed for clinical occlusal adjustment compared to the conventionally fabricated group (MD = -9.10; 95% CI: -15.18 to -3.02; p < 0.001; I² = 96.74%) (Figure 7).

Discussion

In this systematic review and meta-analysis of eight randomized controlled trials, we found that digitally fabricated occlusal splints were not associated with superior pain relief, greater improvement in mouth opening, or higher patient satisfaction compared with conventionally fabricated splints in patients with temporomandibular disorders. By contrast, digital splints were associated with a significant reduction in clinical occlusal adjustment time, suggesting that their main advantage may currently lie in workflow efficiency rather than in clearly superior short-term clinical outcomes.

The most clinically important finding of the present analysis is the absence of a significant difference in pain scores at the last available follow-up. Pain reduction remains the primary therapeutic goal in most patients with TMD [23,24], and the available pooled evidence suggests that both fabrication pathways produce broadly comparable symptomatic outcomes [11,18]. This finding is consistent with the broader TMD literature, in which occlusal splints are generally considered useful conservative tools but not consistently superior to other reversible approaches such as physiotherapy, education, self-management, or multimodal treatment strategies [25,26]. Recent reviews likewise suggest that splints may improve pain and function in selected patients, particularly in the short term, but that superiority across appliance designs or delivery workflows is far from established [27,28].

The lack of a measurable difference in mouth opening further supports the interpretation that the therapeutic effect may depend more on the presence of a properly indicated splint than on the manufacturing technique itself [21]. In routine TMD care, mouth opening is influenced by several factors beyond appliance fabrication, including baseline diagnosis [29], muscular guarding, pain sensitization, parafunctional habits [30], adherence to appliance use, and adjunctive therapies [31]. If both digital and conventional appliances provide similar occlusal coverage, stabilization, and load redistribution, then equivalent functional outcomes would be biologically plausible [32]. From a clinical standpoint, this is reassuring, which suggests that the shift toward digital workflows does not appear to compromise treatment effectiveness in terms of mandibular function.

Patient satisfaction also did not differ significantly between groups, although heterogeneity for this endpoint was high. This likely reflects substantial variability in how satisfaction was defined and measured across studies, as well as differences in patient expectations, splint thickness, fit, esthetics, wear comfort, and the duration of follow-up [13,18,20,21]. Satisfaction is also particularly vulnerable to contextual influences such as chairside communication, adaptation time, and prior treatment experiences [33]. For this reason, although digital workflows are often assumed to improve patient experience through enhanced precision and reproducibility, the currently available randomized data do not demonstrate a consistent advantage in this domain [13,18,20,21].

In contrast, the significantly shorter occlusal adjustment time observed with digitally fabricated splints is both plausible and clinically meaningful. One of the most frequently cited advantages of computer-aided design and manufacturing in prosthodontics and oral rehabilitation is the potential for improved standardization and more predictable fit [34,35]. In the context of TMD appliance therapy, fewer or shorter chairside adjustments may translate into greater efficiency for clinicians, reduced appointment burden for patients, and possibly lower indirect costs [36,37]. This may be particularly relevant in high-volume practices or multidisciplinary TMD clinics where workflow optimization matters [38]. Nevertheless, the high heterogeneity observed for this outcome suggests that the magnitude of this benefit likely depends on the specific digital system used, the operator's experience, printing or milling accuracy, and the adjustment protocol employed in the comparator group. Therefore, while the direction of effect appears favorable to digital fabrication, the exact size of the benefit should be interpreted cautiously.

These findings should also be viewed in the context of current clinical thinking around TMD management. Contemporary guidance generally emphasizes reversible, conservative, and patient-centered interventions as first-line care, including education, behavioral modification, pharmacologic symptom control when needed, physical therapy, and occlusal appliances in selected cases [39]. In this framework, the question is not necessarily whether digital splints are globally better than conventional splints but whether they can deliver equivalent symptom control while improving precision, reproducibility, and efficiency [11]. Our results suggest that this may indeed be the case, as digital splints appear to perform similarly for core patient outcomes while offering a practical chairside advantage.

From a methodological perspective, the substantial heterogeneity seen in pain and satisfaction outcomes deserves attention. This heterogeneity likely reflects differences in patient selection, diagnostic subtypes of TMD, follow-up duration, splint design, fabrication method, and outcome assessment [13,14,20]. TMD is not a single disease entity but a heterogeneous group of conditions, and it is possible that digital splints may perform differently in muscle-related disorders, intra-articular disorders, or mixed phenotypes [40]. Similarly, some studies may have included stabilization splints, whereas others may have differed in thickness, vertical dimension, or wear regimen [13,21,22]. These design differences can dilute pooled estimates and reduce the ability to detect clinically relevant subgroup effects.

Clinical Implications

The findings of this meta-analysis have several implications for future research. First, additional adequately powered randomized trials are needed, with standardized definitions of TMD, splint design, wear protocols, and follow-up time points. Second, future studies should include outcomes that are especially relevant to digital dentistry, such as fit accuracy, remanufacturing reproducibility, time efficiency, cost-effectiveness, and long-term maintenance. Third, patient-centered outcomes should be reported more consistently, ideally using validated measures rather than broadly defined satisfaction scales. Finally, because digital workflows continue to evolve rapidly, the performance of newer CAD/CAM and 3D-printing systems may not be fully captured by the currently available literature. Emerging reports continue to suggest that digitally fabricated appliances are effective, but comparative superiority remains uncertain, which aligns with the results of the present analysis [11,18,22].

Limitations

This study should be interpreted in light of several limitations. The number of included trials was relatively small, and several outcomes were informed by only a limited subset of studies. Heterogeneity was substantial for some endpoints, particularly pain and satisfaction. Variation in splint type, digital workflow, patient selection, and follow-up duration may have influenced the pooled estimates. In addition, some clinically relevant outcomes, such as long-term durability, cost-effectiveness, and the recurrence of symptoms after discontinuation, were either inconsistently reported or unavailable for quantitative synthesis. Nonetheless, by restricting inclusion to randomized data, the present meta-analysis provides the highest level of comparative evidence currently available on this topic.

## Conclusions

Within the available randomized data, the current evidence suggests that digital and conventional splints provide comparable clinical outcomes in the management of TMD with respect to pain reduction, mandibular opening, and patient satisfaction. The main measurable advantage of digitally fabricated splints appears to be reduced occlusal adjustment time, which may support greater clinical efficiency. At present, digital fabrication should probably be viewed not as a clinically superior therapeutic modality but as an alternative workflow that can deliver similar patient outcomes with potential operational benefits.

## Appendices

Table 3 shows the baseline characteristics of the included patients.

**Table 3:** Baseline characteristics of the included patients. SD, standard deviation; NA, not available

Study	Group	Sample size	Age (years), mean (SD)	Male, n (%)	Female, n (%)
Qin et al., 2025 [11]	Digital	63	27.97 (1.22)	11 (17.46%)	52 (82.54%)
	Conventional	63	27.57 (10.42)	13 (20.63%)	50 (79.37%)
Chen et al., 2026 [12]	Digital	66	29.9 (13.55)	24 (36.36%)	42 (63.64%)
	Conventional	66	30.1 (13.70)	20 (30.30%)	46 (69.70%)
Abd Elgaleel et al., 2025 [13]	Digital	6	Range: 20-50	0 (0%)	6 (100%)
	Conventional	6	Range: 20-50	0 (0%)	6 (100%)
Hussein et al., 2025 [14]	Digital	10	Range: 18-45	NA	NA
	Conventional	10	Range: 18-45	NA	NA
Pai et al., 2025 [18]	Digital	12	29.4 (7.1)	4 (33.33%)	8 (66.67%)
	Conventional	12	29.4 (7.1)	4 (33.33%)	8 (66.67%)
Algabri et al., 2017 [20]	Digital	15	30 (NA)	2 (13.3%)	13 (86.7%)
	Conventional	15	30.4 (NA)	4 (26.7%)	11 (73.3%)
Berntsen et al., 2018 [21]	Digital	7	NA	NA	NA
	Conventional	7	NA	NA	NA
Pho Duc et al., 2016 [22]	Digital	16	26.8 (3.3)	6 (37.5%)	10 (62.5%)
	Conventional	16	30.5 (9.2)	4 (25%)	12 (75%)
